# Application of Silver Nanostructures Synthesized by Cold Atmospheric Pressure Plasma for Inactivation of Bacterial Phytopathogens from the Genera *Dickeya* and *Pectobacterium*

**DOI:** 10.3390/ma11030331

**Published:** 2018-02-25

**Authors:** Anna Dzimitrowicz, Agata Motyka, Piotr Jamroz, Ewa Lojkowska, Weronika Babinska, Dominik Terefinko, Pawel Pohl, Wojciech Sledz

**Affiliations:** 1 Department of Analytical Chemistry and Chemical Metallurgy, Faculty of Chemistry, Wroclaw University of Science and Technology, 27 Wybrzeze Wyspianskiego, 50-370 Wroclaw, Poland; piotr.jamroz@pwr.edu.pl (P.J.); terefinko.dominik@gmail.com (D.T.); pawel.pohl@pwr.edu.pl (P.P.); 2 Department of Biotechnology, Intercollegiate Faculty of Biotechnology University of Gdansk and Medical University of Gdansk, University of Gdansk, 58 Abrahama, 80-307 Gdansk, Poland; agata.motyka@biotech.ug.edu.pl (A.M.); ewa.lojkowska@biotech.ug.edu.pl (E.L.); weronikababinska29@gmail.com (W.B.); wojciech.sledz@biotech.ug.edu.pl (W.S.)

**Keywords:** nanoparticles, atmospheric pressure glow discharge, plasma-liquid interactions, pectinolytic bacteria, plant protection, soft rot, blackleg

## Abstract

Pectinolytic bacteria are responsible for significant economic losses by causing diseases on numerous plants. New methods are required to control and limit their spread. One possibility is the application of silver nanoparticles (AgNPs) that exhibit well-established antibacterial properties. Here, we synthesized AgNPs, stabilized by pectins (PEC) or sodium dodecyl sulphate (SDS), using a direct current atmospheric pressure glow discharge (dc-APGD) generated in an open-to-air and continuous-flow reaction-discharge system. Characterization of the PEC-AgNPs and SDS-AgNPs with UV/Vis absorption spectroscopy, transmission electron microscopy, energy dispersive X-ray spectroscopy, and selected area electron diffraction revealed the production of spherical, well dispersed, and face cubic centered crystalline AgNPs, with average sizes of 9.33 ± 3.37 nm and 28.3 ± 11.7 nm, respectively. Attenuated total reflection-Fourier transformation infrared spectroscopy supported the functionalization of the nanostructures by PEC and SDS. Antibacterial activity of the AgNPs was tested against *Dickeya* spp. and *Pectobacterium* spp. strains. Both PEC-AgNPs and SDS-AgNPs displayed bactericidal activity against all of the tested isolates, with minimum inhibitory concentrations of 5.5 mg∙L^−1^ and 0.75–3 mg∙L^−1^, respectively. The collected results suggest that the dc-APGD reaction-discharge system can be applied for the production of defined AgNPs with strong antibacterial properties, which may be further applied in plant disease management.

## 1. Introduction

Nanoparticles (NPs) exhibit unique thermal, chemical, optical, and mechanical [1] properties in comparison to the corresponding macroscopic scale forms. Their noteworthy features are related to their high surface to volume ratio that increases as the average size of the NPs decrease. Hence, nanostructures of various chemical compositions, including silver nanoparticles (AgNPs), have found countless applications in the medical, pharmaceutical, engineering, energy, and food production sectors, among others. To meet the requirements of such diverse industries, numerous methods involving chemical (i.e., photo-, thermo-, sono-, and electrochemical), physical (i.e., laser ablation), and biological (i.e., green synthesis) procedures have been used in order to obtain stable-in-time AgNPs [2]. Recently, special emphasis has been placed on biological synthesis methods, as they require neither toxic reducing or capping agents nor radioactive irradiation. The biological synthesis procedures instead take advantage of plant extracts or essential oils as the reducing and stabilizing compounds, or involve soil microorganisms that are capable of synthesizing AgNPs from an appropriate precursor [1,3,4].

In our opinion, cold atmospheric pressure plasmas (CAPPs)-based AgNPs synthesis methods are a tempting alternative to the techniques described above. Namely, these procedures receive substantial interest owing to their one-stage process, simplicity, and lack of requirements for toxic or irritating reducing agents. The treatment of liquids with CAPPs results in elevated concentrations of reactive oxygen species (ROS), reactive nitrogen species (RNS), and hydrated electrons in the solution, which are then involved in the reduction reactions with the precursors of the AgNPs [5,6]. Several research groups have investigated the usage of CAPPs-based processes for AgNPs production [5,6,7,8]. However, the vast majority of the previously reported CAPPs-based reaction-discharge systems work in a non-flowing, stationary mode, and are constructed, for example, with the use of bulky liquids as electrodes. Such CAPPs-based systems exhibit several limitations. These include uncontrolled interactions of the plasmas with the AgNPs precursor solutions. Therefore, we have previously developed a reaction-discharge system for the continuous synthesis of Ag nanostructures [9,10], based on direct current atmospheric pressure glow discharge (dc-APGD) sustained in an open-to-air atmosphere [11]. In that reaction-discharge system, dc-APGD was operated between the surface of a flowing liquid anode (FLA) and a pin-type tungsten cathode, and was subsequently used for the synthesis of raw-AgNPs and gelatine (GEL)-stabilized AgNPs [11]. The broad spectrum antimicrobial activities of Ag(I) ions is well-documented [12]. Additionally, there is a growing relevance of nanostructures within the agricultural sector due to their numerous applications as: direct antimicrobial agents; delivery systems providing diminished amounts of active pesticides, fertilizers, or genetic material of interest; nanosensors of plant pathogens or pesticide residuals; and, nanocatalysts established for degradation of the remaining chemical pesticides [1,13].

Therefore, we postulate that AgNPs might also be an effective method for the control of pectinolytic plant pathogenic bacteria from the genera *Dickeya* and *Pectobacterium*. The high economic importance of these microorganisms was emphasized by Mansfield et al. (2012), who listed them among the top ten plant pathogenic bacteria [14]. Notably, *Dickeya* spp. and *Pectobacterium* spp. are the causative agents of blackleg on potato and soft rot on many crops, vegetables, and ornamentals. Common symptoms of blackleg involve the softening and blackening of the stem base and the presence of wilting leaves, while soft rot refers to maceration of the plant inner tissue [15]. These diseases result from the ability of *Dickeya* spp. and *Pectobacterium* spp. to secrete plant cell wall-degrading enzymes, including pectinases and cellulases [16], whose actions disrupt the integrity of plant cells, leading to spillage of valuable nutrients from the apoplasts. Taking into account that these phytopathogens are widespread in different climatic zones [17] and trigger disease symptoms in plant species from at least 35% of the angiosperm orders [18], it is of crucial importance to control their occurrence and transmission pathways. Previously, approaches that are based mainly on the avoidance of plant contamination, implementation of seed testing programs, or the application of hygienic procedures have been proposed to remove plant pathogenic bacteria from the crop production pipelines [19]. For instance, physical (hot water, steam, dry hot air, solar, UV irradiation) and chemical (antibiotics, natural bactericides, synthetic antimicrobial peptides) treatments were tested in order to reduce the load of *Dickeya* spp. and *Pectobacterium* spp. on potato tubers [20]. It is worth to stress that no method enables the eradication of plant pathogenic bacteria post infection, thus only preventive measures are commonly applied to limit their further spread [19].

The main objective of the current work was to apply dc-APGD operated in the gap between the surface of the FLA and the pin-type tungsten cathode, working in a continuous-flow mode, for the production of high amounts of size- and shape-defined AgNPs that are stabilized either by pectins (PEC-AgNPs) or by sodium dodecyl sulphate (SDS-AgNPs). The antimicrobial properties of these AgNPs were also tested against *Dickeya* spp. and *Pectobacterium* spp. To the best of our knowledge, this is the first work in which PEC-AgNPs and SDS-AgNPs synthesized via a dc-APGD-based method have been proposed as antimicrobials to eradicate plant pathogens of high economic importance.

## 2. Results

### 2.1. Characterization of the PEC-AgNPs and SDS-AgNPs

#### 2.1.1. Optical Properties of the Ag Nanostructures

**Figure 1:**
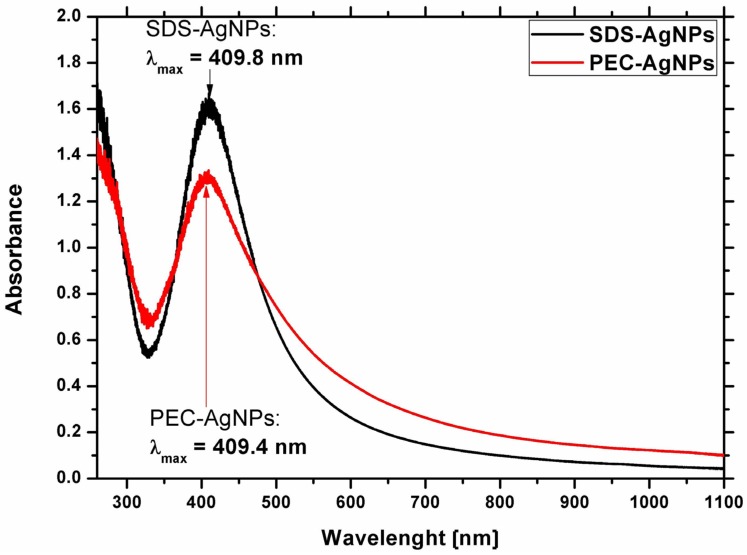
UV/Vis absorption spectra of Ag nanostructures, stabilized by pectins (PEC) (red line) or sodium dodecyl sulphate (SDS) (black line).

To examine possibility of using dc-APGD for the production of stable-in-time AgNPs, the optical properties of the obtained colloidal suspensions of Ag nanostructures were investigated. Following dc-APGD treatment, the colours of the solutions became yellowish, which is typical for solutions containing AgNPs [11]. In order to confirm the production of Ag nanostructures, UV/Vis measurements were conducted. In general, the λ_max_ of the collective oscillating electric fields, referred to as the Localized Surface Plasmon Resonance (LSPR) absorption band for AgNPs, is situated in the range of 400–750 nm [11]. The λ_max_ of the LSPR absorption band for PEC-AgNPs was located at 409.4 nm, while for SDS-AgNPs, the λ_max_ of the LSPR absorption band was observed at 409.8 nm (Figure 1).

The shift in the position of the λ_max_ of the LSPR absorption band towards red wavelengths, as in the case of SDS-AgNPs in comparison to PEC-AgNPs, might indicate the production of larger AgNPs based on Mie’s light scattering theory [21]. Furthermore, the shapes of both the LSPR absorption bands were symmetrical. This was indicative of the formation of monodisperse and uniform in size Ag nanostructures, following the CAPP treatment [21].

#### 2.1.2. Morphology of the Ag Nanostructures

**Figure 2:**
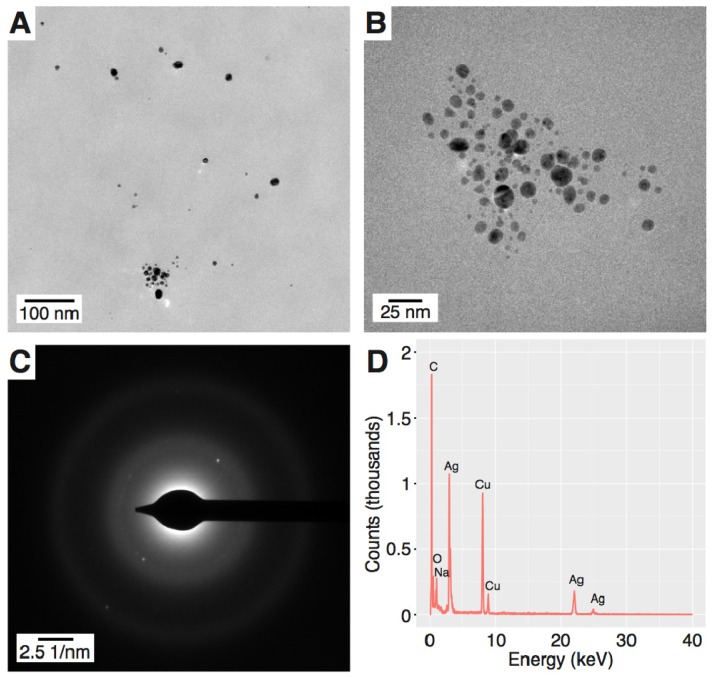
Morphology of the produced PEC-silver nanoparticles (AgNPs). (**A**,**B**) Representative transmission electron microscopy (TEM) images; (**C**) the selected-area electron diffraction (SAED) pattern; and (**D**) the energy dispersive X-ray spectroscopy (EDX) spectrum.

**Figure 3:**
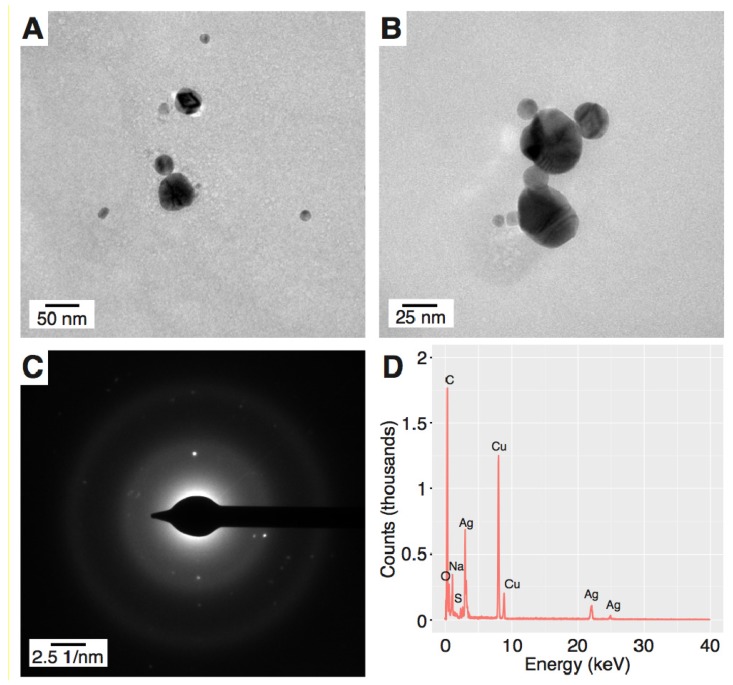
Morphology of the produced SDS-AgNPs. (**A**,**B**) Representative TEM images; (**C**) the SAED pattern; and (**D**) the EDX spectrum.

To reveal the granulometric properties of the resultant Ag nanostructures, transmission electron microscopy (TEM) assisted with selected-area electron diffraction (SAED) and energy dispersive X-ray spectroscopy (EDX) were used. According to the TEM visualization, PEC-AgNPs had a spherical shape and an average size of 9.33 ± 3.37 nm (Figure 2A,B). Similarly, SDS-AgNPs were well-dispersed, but with a larger average size of 28.3 ± 11.7 nm (Figure 3A,B) and a wider size distribution than PEC-AgNPs. In order to define the crystalline structure of the synthesized AgNPs, SAED analyses were performed. The d-spacings were calculated based on Bragg’s law and the position of the peaks on the selected area diffraction pattern (SADP). The d-spacings of the PEC-AgNPs were determined to be 2.315, 2.232, 1.852, and 1.190 Å (Figure 2C), which correspond to the Miller indices of (111), (200), (220), and (311), respectively [22]. In terms of the SDS-AgNPs, the d-spacings values were determined to be 2.315, 2.100, 1.600, and 1.212 Å (Figure 3C), indicating Miller indices of (111), (200), (220), and (311), respectively [22]. Based on these Miller indices and the work of Khan et al. [23], both the PEC-AgNPs and the SDS-AgNPs were determined to be face cubic centered (fcc) crystalline structures. Finally, EDX was applied to determine the elemental compositions of the AgNPs. As shown in Figure 2D and Figure 3D for PEC-AgNPs and SDS-AgNPs, respectively, peaks corresponding to metallic Ag were detected. Furthermore, the EDX spectrum of the SDS-AgNPs revealed the presence of sulphur, which is part of the chemical structure of SDS, the capping agent. Moreover, copper and carbon were seen in both samples due to the use of copper grids for imposing the samples prior to the TEM, SAED, and EDX analyses, and to the chemical compositions of the capping agents, respectively.

#### 2.1.3. Surface Functionalization of the Ag Nanostructures

**Figure 4:**
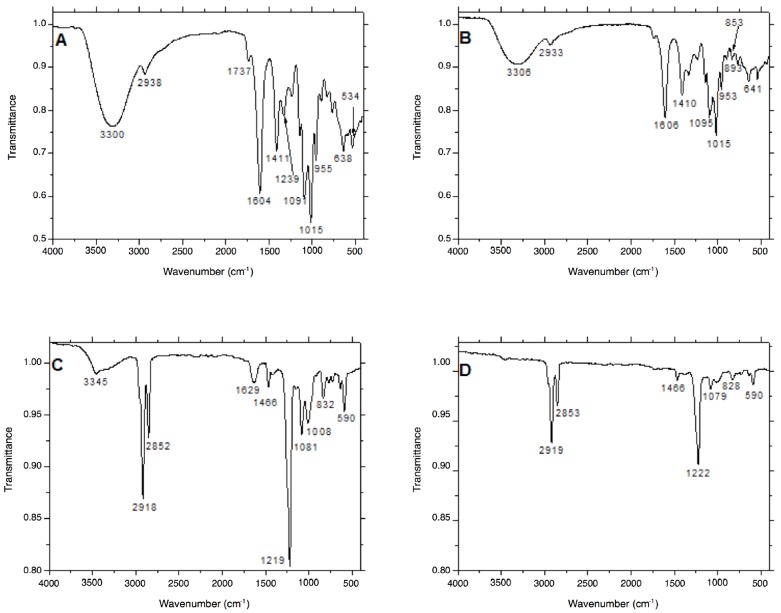
Comparison of the ATR FT-IR spectra acquired for: (**A**) 0.5% PEC solution before dc-APGD treatment; (**B**) solution containing PEC-AgNPs; (**C**) 0.5% solution of SDS before dc-APGD treatment, and (**D**) solution containing SDS-AgNPs.

Interactions between the surfaces of the AgNPs and either PEC or SDS were evaluated by applying attenuated total reflection-Fourier transformation infrared spectroscopy (ATR FT-IR). By comparing the ATR FT-IR spectra of PEC before dc-APGD treatment and PEC-AgNPs after the impact of the CAPP, no change in the chemical composition of PEC was observed (Figure 4A). Focusing on the ATR FT-IR spectrum of PEC prior to dc-APGD treatment, a band at 3300 cm^−1^, referring to the stretching vibrations of the ‒OH group, was detected, and the band at 2938 cm^−1^ was assigned to the stretching vibrations of the CH_2_ group [24]. In addition, the presence of the stretching vibrations of the C=O group was determined based on the presence of bands at 1737, 1604, and 1411 cm^−1^, while an asymmetric deformation of the C‒O‒C group was noted based on the band at 1235 cm^−1^ [24]. Additionally, the presence of C=C double bonds in the chemical structure of PEC was confirmed by the observation of a band at 1015 cm^−1^. Moreover, bands situated at 955, 893, and 825 cm^−1^, indicative of the wagging vibrations of the CH_2_ group, were recorded [3]. In the ATR FT-IR spectrum of the PEC-AgNPs following dc-APGD treatment, the low intensity of the stretching vibrations of ‒OH (associated with the band at 2933 cm^−1^) and of the C=O groups might suggest the formation of hydrogen bonds between the surface of AgNPs and PEC (Figure 4B). Not surprisingly, slight shifts in the position of the vibrations for PEC-AgNPs and PEC were detected, which is consistent with the formation of hydrogen bonds between the synthesized Ag nanostructures and the stabilizer.

Based on the ATR FT-IR spectrum of SDS before and after the dc-APGD treatment, the chemical structure of this stabilizer appeared to be retained (Figure 4C). An intense band at 3455 cm^−1^ corresponding to ‒OH stretching vibrations was detected in the chemical structure of SDS before the influence of dc-APGD, as were bands at 2918 and 2852 cm^−1^, which are associated with the C‒H stretching vibrations [25,26]. Furthermore, bands localized at 1629, 1466, and 1417 cm^−1^ were detected, which refer to the asymmetric deformation of the terminal CH_3_ group [27]. C‒O symmetric vibrations, corresponding to the bands localized at 1147, 1081, and 1008 cm^−1^ [28], and the SO_2_ asymmetric vibrational feature, corresponding with the band at 1219 cm^−1^ [27], were also identified. According to the ATR FT-IR spectrum of SDS-AgNPs after dc-APGD (Figure 4D), we postulate that the conjugation of SDS to the surface of the AgNPs was successful based on the absence of the ‒OH stretching vibrations (the band situated at 3345 cm^−1^ before CAPP treatment), and with the asymmetric deformation of the terminal CH_3_ group corresponding to the band located at 1629 cm^−1^. In summary, these results suggest that the surfaces of the AgNPs have been functionalized by SDS through coordination bonds between the Ag and S.

### 2.2. Concentration of the Ag Nanostructures

Flame atomic absorption spectrometry (FAAS) was applied to assess the concentrations of the purified PEC-AgNPs and SDS-AgNPs following purification of the AgNPs via dialysis. Concentrations of 11.0 mg∙L^−1^ and 24.0 mg∙L^−1^ of Ag, respectively, were determined. When considering that the initial amount of Ag(I) ions was 200 mg∙L^−1^, approximately 5.6% and 12% of the precursor Ag was recovered as PEC-AgNPs and SDS-AgNPs, respectively.

### 2.3. Antibacterial Properties of the Ag Nanostructures against Dickeya spp. and Pectobacterium spp.

The antibacterial properties of the Ag nanostructures were tested against five strains of bacteria from the genera *Dickeya* and *Pectobacterium*, namely: *Dickeya solani* IFB0099 (Dsol), *Pectobacterium atrosepticum* IFB5103 (Pba), *Pectobacterium carotovorum* subsp. *brasiliense* IFB5390 (Pcbr), *Pectobacterium carotovorum* subsp. *carotovorum* IFB5118 (Pcc), and *Pectobacterium parmentieri* IFB5308 (Ppa) (Table 1).

Both PEC-AgNPs and SDS-AgNPs exhibited antibacterial activities against all of the tested strains. Notably, SDS-AgNPs showed higher effectiveness with minimal inhibitory concentrations (MICs), ranging from 0.75 to 3 mg∙L^−1^ in comparison to PEC-AgNPs, for which the MICs equalled 5 mg∙L^−1^ for all of the isolates. In general, the different species responded homogeneously to the applied treatment, with the exception of Pba that presented a higher susceptibility to the SDS-AgNPs than the other isolates. For all of the investigated strains and in the concentration ranges analysed, the minimal bactericidal concentrations (MBCs) were identical to the established MICs, which indicated a bactericidal, not bacteriostatic, action of the PEC-AgNPs and SDS-AgNPs.

## 3. Discussion

In the era of rapidly spreading antibiotic resistance among microorganisms, there is a high demand for efficient eradication methods effective against multiple bacterial species, and for which the emergence of resistant strains would be unlikely. A promising approach might be the application of AgNPs as direct antimicrobials, as their effectiveness have been demonstrated against approximately 650 bacterial species, as well as many fungi, viruses, and yeasts [29]. Over 250 consumer products belonging to diverse sectors take advantage of the unique properties of AgNPs [29]; therefore, their more extensive usage within plant disease management is only a matter of time.

With future agricultural or horticultural applications in mind, it is essential to select a nanostructures synthesis method that does not require toxic or irritating substances that are often used as reducing agents (e.g., sodium borohydride, *N*-*N*-dimethylformamide [30]) or stabilizers (e.g., sodium citrate, polyvinylpyrrolidone [31]). Such compounds lower the biosafety, biodegradability, and biocompatibility of the obtained AgNPs, limiting their introduction into biological systems. In this regard, a suitable solution is the utilization of a CAPPs-based AgNPs production method, as reported here. This procedure allows for the generation of spherical NPs in an effortless, cost-effective, and environmentally friendly one-stage process. An advantage of this synthesis approach is the generation of ROS, RNS, and hydrated electrons in the plasma-liquid interface, which trigger the reduction of the AgNPs precursor, ensuring no need for chemical reducing agents [4,9,10,11]. Additionally, the production rate of AgNPs is strongly increased by the continuous flow of the precursor solution within the designed reaction-discharge system. Thus, the CAPPs-based AgNPs production method is a tempting alternative to the other techniques applied so far.

In order to obtain non-aggregated, uniform in size and shape Ag nanofluids, it is necessary to include a stabilizer in the reaction mixture to prevent the uncontrolled growth and sedimentation of the nanostructures. In the present work, we examined two capping agents, PEC and SDS, in relation to their influence on the size, shape, and antimicrobial properties of the resultant AgNPs. PEC is a key heteropolysaccharide that is present in the primary cell wall of terrestrial plants. *Dickeya* spp. and *Pectobacterium* spp. secrete pectinases that break down this polymer into pectic oligomers [16]. Products of PEC cleavage can then be assimilated via the TogMNAB transport system of the pathogen [32]. In our study, we produced PEC-AgNPs with the expectation of a high rate of release of Ag(I) ions after partial digestion of the PEC stabilizer by the targeted plant pathogen. As the liberated oligogalacturonides trigger strong chemotaxis of the neighbouring *Dickeya* spp. and *Pectobacterium* spp. cells via the TogB receptor [32], we also aimed for efficient attraction of the remaining bacteria to the site of the AgNPs action. We attribute the excellent stabilizing properties of PEC with the high molecular weight (>10^4^ g∙mol^−1^) of this polymer. This provides steric stabilization related to the spatial dimension of the molecular compounds proportional with the range of the London’s forces of attraction [33]. The second stabilizer used was SDS, which is a non-ionic surfactant of broad-spectrum antimicrobial activity. Being an easily available and low-cost detergent, SDS is often utilized to lyse bacterial cells in, for example, DNA and RNA-isolation procedures. Moreover, SDS displays low harmfulness towards humans, and is included, for instance, in toothpastes. Concerning the AgNPs stabilizing properties of SDS, this heteropolar surfactant absorbs to the surfaces of the produced AgNPs, limiting aggregation of the NPs [34].

In the present work, we showed that PEC-AgNPs and SDS-AgNPs are effective antimicrobials against various *Dickeya* spp. and *Pectobacterium* spp. The exact antibacterial mechanism of AgNPs is not yet fully understood. The most prominent theory relies on the combined role of the released Ag(I) ions and the AgNPs themselves. These antimicrobials are thought to interact with thiol, carboxyl, hydroxyl, amino, phosphate, and imidazole groups that are located on bacterial membranes, leading to membrane deformations and cell wall collapse [35]. As a result, entry points into the bacterial cells appear, allowing for further penetration of the nanofluid. Subsequent interactions with the intracellular enzymes and DNA inhibit bacterial replication, resulting in cell death [35].

Previously, AgNPs synthesized by various methods have been applied as direct antimicrobials, mostly against human pathogens (e.g., *Staphylococcus aureus*, *Salmonella enterica*, *Klebsiella pneumoniae*, *Pseudomonas aeruginosa*, *Escherichia coli* [36,37]) and animal-affecting infectious agents (e.g., *Aspergillus fumigatus*, *Vibrio alginolyticus*, *Aeromonas hydrophila*, *Edwardsiella tarda*, *Saprolegnia* spp. [38,39]). Much less attention has been given to phytopathogens, though AgNPs of diverse formulations were shown to be potent against plant pathogenic fungi, such as *Bipolaris sorokiniana*, *Magnaporthe grisea* [40], *Colletotrichum* spp. [41], *Fusarium oxysporum* [42], or *Alternaria alternata* [43]. In terms of plant pathogenic bacteria, Park et al. (2006) defined the effective concentration of nanosized silica-silver against *Pseudomonas syringae* pv. *syringae* and *Xanthomonas campestris* pv. *vesicatoria* as 100 mg∙L^−1^, which is 10-fold higher than the required concentrations for antifungal activity [44]. An innovative approach was proposed by Ocsoy et al. (2013), who reported an excellent activity of 16 mg∙L^−1^ for DNA-directed AgNPs on graphene oxide against *Xanthomonas perforans* [35]. Perhaps the need for higher concentrations or more specialized nanocomposites accounted for the low interest in the application of AgNPs against bacterial phytopathogens. Interestingly, Chua et al. (2012) observed that *Arabidopsis thaliana* is more resistant to *Pseudomonas syringae* pv. *tomato*, following pretreatment with a nanosized Ag–silica hybrid complex [45]. To the best of our knowledge there is only one previous report on the application of AgNPs (in that case, synthesized with the use of oak leaf and fruit extracts) on any of the pectinolytic plant pathogenic bacteria that we tested [46]. Unfortunately, the high inter-strain phenotypic variability [47] and the lack of information on the concentration of the AgNPs used in that study prevents a comparison of our work with their study. In our work, the MICs and MBCs against the investigated pectinolytic bacteria were 0.75–3 mg∙L^−1^ and 5 mg∙L^−1^ for SDS-AgNPs and PEC-AgNPs, respectively. In general, these AgNPs concentrations were lower than those that were previously reported for plant pathogenic bacteria [35,44], but higher than the ones that have been efficiently applied against phytopathogenic fungi [42,44].

The synthesis method, capping agents used, and the size and shape influence the unique features of NPs. In our case, spherical PEC-AgNPs and SDS-AgNPs of approximately 9.33 ± 3.30 nm and 28.3 ± 11.8 nm, respectively, were synthesized. Interactions of AgNPs with soil constituents, treated plants, and natural microbiota need to be taken into consideration when thinking about future agricultural applications [29]. The physico-chemical properties of soil (including texture, pH, cations exchange capacity, and organic matter) and nanostructures affect the dissolution, agglomeration, and aggregation of the NPs, which strongly impacts their further mobility and sorption [48]. AgNPs aggregates exhibit lower bioavailability, and, consequently, diminished antibacterial properties, emphasizing the importance of a proper NPs stabilizer. In terms of natural soil microbiota [49,50], the highest level of toxicity of AuNPs against nitrifying bacteria, as a model microorganisms in this kind of research, was shown to be for AgNPs < 5 nm in size [51]. Hence, the environmental impact of the herein reported larger in size PEC-AgNPs and SDS-AgNPs is predicted to be rather limited. Regarding the effects on plants, the plant species and state of growth, the environmental conditions, and the AgNPs concentration, properties, and mode of application appear to be of crucial significance [29]. Based on our previous studies on the impact of exogenous caffeine application on plant growth and development [52] and the studies of Lee et al. (2012) [53], it is expected that there will be lower bioavailability of Ag(I) ions from AgNPs in soil in comparison to the agar medium, which will result in diminished accumulation of Ag(I) ions within the plant biomass. Similarly to what has been stated for soil microbiota, smaller in size NPs are considered to cause greater cellular, physiological, and genotoxicity in plants [29]. On the other hand, there are many reports on the beneficial influence of Ag nanostructures on plant growth and development in soil. For example, stimulating effects of 10–30 nm AgNPs (concentrations 20, 40, and 60 ppm) on *Phaseolus vulgaris* and *Zea mays* in terms of root/shoot elongation, leaf surface area, carbohydrates, proteins, and chlorophyll contents were observed [54]. Also, Vannini et al. (2013) noted root elongation in *Eruca sativa* if the applied AgNPs concentration was within 0.1–20 mg∙L^−1^ [55].

We assume that the optimized use of well-studied, stable, and eco-friendly AgNPs showing antibacterial properties can have an advantageous influence on plants and therefore encourage further agricultural applications. In the future, demand for crops yield is predicted to rapidly increase, while natural resources, like land, water, and soil fertility remain limited. Constant concerns that are associated with modern intensive farming practices resulting from the Green Revolution focus on the environmental impact of fertilizers and pesticides, highlighting the necessity of more sustainable protection methods [56]. Several advantages have been attributed to nanoformulations relative to, for instance, biopesticides. These include no requirements for shelf life, high stability in the field, no effect of a fluctuating environment, high surface area and coverage, and very low dosage [29]. In 2011, there were three thousand patent applications dealing with the topic of nanofluids, implying that this approach might revolutionize the agricultural sector in the forthcoming years [57].

The herein reported PEC-AgNPs and SDS-AgNPs, with antibacterial properties against *Dickeya* spp. and *Pectobacterium* spp., could play a role in integrative plant protection and may be considered as substitutes for compounds, such as hydrogen peroxide, being currently used for the disinfection of packaging materials and agricultural equipment. It is expected that it will be possible to scale up the described continuous-flow reaction-discharge system for the production of larger quantities of AgNPs. This can be done, for instance, through increasing the diameters of both electrodes. Keeping the same linear input flow rate of the AgNPs precursor solution, i.e., 28 cm∙min^−1^, a five-fold increase in the diameter of the quartz-graphite tube in the cathodic compartment would require a 25-fold increase in the flow rate of the mentioned solutions. Or, in other words, a flow rate of 88 mL∙min^−1^ would be obtained with a graphite tube of 20 mm, instead of the current 3.5 mL∙min^−1^ flow rate obtained using a 4 mm diameter graphite tube. To provide the same 45-mA discharge current at the same 5-mm discharge gap, the voltage would certainly be higher than 1.1 kV as the resistively of the system would increase. However, it should be noted that the magnitude of this increase would be related to the voltage-current characteristics of the whole reaction-discharge system, which is not linear since it strongly depends on the resistively of the discharge column.

## 4. Materials and Methods

### 4.1. Reagents and Solutions

All of the reagents that are used for the syntheses of PEC-AgNPs and SDS-AgNPs were of analytical grade or higher purity. A working solution of 200 mg∙L^−1^ of Ag(I) ions was prepared by dissolving 0.0315 g of solid silver nitrate (AgNO_3_, Avantor Performance Materials, Gliwice, Poland) in 100 mL of re-distilled water. To prevent uncontrolled growth, aggregation, and sedimentation of the synthesized AgNPs, PEC (Agdia-Biofords, Evry Cedex, France) or SDS (Avantor Performance Materials, Gliwice, Poland) was added to the working solution of the Ag nanostructures precursor to a final concentration as indicated. Re-distilled water was used throughput.

### 4.2. Synthesis of PEC-AgNPs and SDS-AgNPs in the dc-APGD-Based Reaction-Discharge System

**Figure 5:**
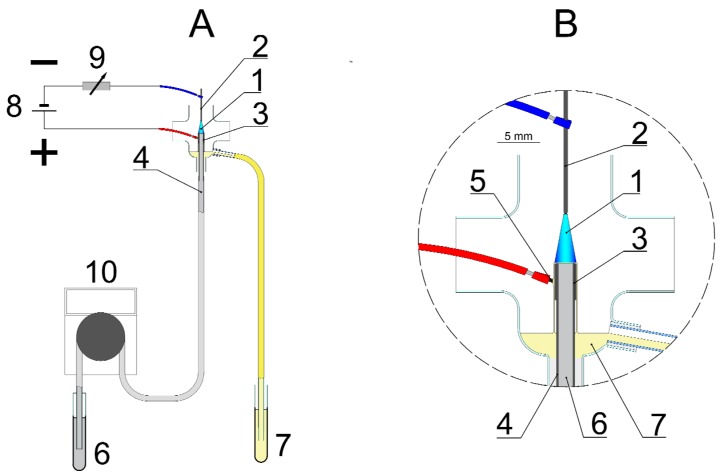
A scheme of the flowing liquid anode (FLA)-atmospheric pressure glow discharge (APGD) reaction-discharge system for the continuous-flow production of Ag nanostructures. 1—dc-APGD; 2—pin-type metallic cathode; 3—graphite tube; 4—quartz capillary; 5—platinum wire; 6—flowing liquid anode solution; 7—solution after the dc-APGD treatment; 8—dc-HV generator; 9—ballast resistor; 10—peristaltic pump. (**A**) reaction-discharge system for the continuous synthesis of Ag nanostructures; (**B**) enlarged reaction-discharge system in circle on the right.

The production of PEC-AgNPs as well as SDS-AgNPs was performed in the dc-APGD-based reaction-discharge system described previously by Dzimitrowicz et al. (2017) for the size-controlled synthesis of raw-AgNPs and GEL-AgNPs [11]. Briefly, in the continuous-flow reaction-discharge system (Figure 5), dc-APGD (1) was operated in the 4.0 mm gap between the surface of the flowing liquid solution, acting as the anode (6), and a pin-type tungsten electrode (2), acting as the cathode [11].

Both of the electrodes were placed in a quartz chamber and sustained by a dc-HV potential (~1100 V) from a dc-HV generator (8, Dora Electronics Equipment, Wroclaw, Poland). There were four holes in the quartz chamber in order to: (a) insert the pin-type tungsten electrode, (b) introduce the working solution containing the AgNPs precursor and the capping agent, (c) collect the colloidal suspensions after the impact of dc-APGD, and (d) provide the open-to-air discharge atmosphere. The electrical contact was supplied by a Pt wire (5) connected to a graphite tube (3) mounted onto the quartz capillary (4). A constant value of the discharge current, i.e., 45 mA, was maintained by applying a 10 kΩ ballast resistor (9, Tyco Electronics, Berwyn, IL, USA). To synthesize a colloidal suspension of AgNPs, a working solution, consisting of 200 mg∙L^−1^ of Ag(I) ions and one of the above-mentioned capping agents (SDS or PEC), was introduced to the reaction-discharge system at a flow rate of 3.5 mL min^−1^ via a quartz-graphite tube. A four channel peristaltic pump (10, MasterFlex L/S, Cole-Parme^®^, Vernon Hill, IL, USA) was used to deliver the working solution to the proposed reaction-discharge system. Subsequently, the dc-APGD was ignited. The mentioned working conditions of the reaction-discharge system were chosen as those in which a stable dc-APGD would be operated.

To find the optimal conditions for the synthesis of spherical AgNPs in the described reaction-discharge system, three concentrations of PEC and SDS, i.e., 0.10, 0.25, and 0.50% (*m*/*v*), and three final concentrations of the Ag(I) ions, i.e., 50, 100, or 200 mg∙L^−1^, were analyzed. A symmetrical LSPR absorption band was acquired only when the highest concentration of the stabilizers (0.5% (*m*/*v*)) and the highest concentration of the Ag(I) ions (200 mg∙L^−1^) were used. These conditions were applied in the subsequent experiments. The use of higher concentrations (>1% (*m*/*v*)) of both stabilizers in the solutions of AgNPs was avoided in order to protect the pin-type tungsten electrode, which was covered with a white coatings of these substances, leading to inconvenient changes in the voltage-current characteristics of dc-APGD. The post-dc-APGD working solutions were collected in sterile 10 mL polypropylene vials (7). All the AgNPs solutions were kept for further analyses at 4 °C.

### 4.3. Characterization of the PEC-AgNPs and SDS-AgNPs

To reveal the optical properties of the colloidal suspensions of PEC-AgNPs and SDS-AgNPs, UV/Vis absorption spectra were acquired with a step of 0.2 nm and in the range from 300 to 1100 nm, using a double-beam UV/Vis Specord 210 spectrophotometer (Analytic Jena, Jena, Germany). The UV/Vis absorption spectra were recorded 24 h after the dc-APGD treatment. It was previously determined that after 24 h, the position of LSPR absorption band does not change for at least five weeks [11]. Re-distilled water was used to zero the instrument. Granulometric properties of the obtained AgNPs were determined according to their size, shape, and crystalline structure by high resolution TEM (FEI Tecnai G2 20 X-TWIN, FEI, Hillsboro, OR, USA), equipped with EDX and SAED systems (AZtecEnenrgy, Oxford Instruments, Abingdon, UK). To examine the morphology of the AgNPs, one drop of the colloidal suspension of AgNPs was put onto a copper grid (CF 400 Cu-UL, GF MICROSYSTEMS, Poznan, Poland) and dried by evaporation after IR lamp (R95E, Philips Lighting, Pila, Poland) irradiation. The size and shape distributions of the synthesized PEC-AgNPs and SDS-AgNPs were assessed based on the measurements of the diameters of 50 NPs. To establish the size distribution of the synthesized AgNPs, the FEI Software (version 3.2 SP6 build 421, FEI, Hillsboro, OR, USA) was applied. Subsequently, ATR FT-IR was used to confirm functionalization of the AgNPs by the desired stabilizer (PEC or SDS). The ATR FT-IR spectra were acquired for solutions consisting of 200 mg∙L^−1^ of Ag(I) ions and either 0.5% (*m*/*v*) of PEC or 0.5% (*m*/*v*) of SDS, before and after the impact of the dc-APGD. The ATR FT-IR spectra were recorded in the spectral range from 4000 to 400 cm^−1^ with a scan speed of 64 scans and resolution of 4 cm^−1^ via a Vertex 70v ATR FT-IR spectrometer (Bruker, Bremen, Germany). The ATR FT-IR analyses were performed separately on drops of colloidal suspensions of PEC-AgNPs and SDS-AgNPs that had been placed onto a diamond ATR cell and evaporated under vacuum conditions.

### 4.4. Purification of PEC-AgNPs and SDS-AgNPs from Ag(I) Ions

The dialysis method was applied to purify the AgNPs from the remaining unconverted Ag(I) ions. Ten mL of the synthesized PEC-AgNPs or SDS-AgNPs solution was transferred to a dialysis tube (MWCO = 14,000, Sigma-Aldrich, Poznan, Poland), which was then immersed in 200 mL of re-distilled water. In order to completely remove the residual Ag(I) ions, the dialysis was performed for 24 h and was conducted with mixing on a magnetic laboratory stirring (1000 rpm, WIGO, Pruszkow, Poland), as was done previously [58].

### 4.5. Determination of the Production Efficiency of the Purified PEC-AgNPs and SDS-AgNPs

The concentrations of the purified PEC-AgNPs and SDS-AgNPs were determined using single-beam flame atomic absorption spectrometer (FAAS, Waltham, MA, USA) supported with a deuterium lamp, after the investigated solutions were digested with 65% (m/m) HNO_3_ (Avantor Performance Materials, Poland) and heated to a boil for 30 min.

### 4.6. Bacterial Strains and Their Growth Conditions

The phytopathogens used in this study are described in Table 2.

All of the isolates originate from the collection of plant pathogenic bacteria of the Intercollegiate Faculty of Biotechnology University of Gdansk and Medical University of Gdansk (IFB UG & MUG). The strains were previously stored at −80 °C in 40% glycerol. They were recovered by plating on Tryptone Soya Agar medium (TSA, Oxoid, Hampshire, UK), followed by 24 h incubation at 28 °C. A single bacterial colony of each strain was collected from a TSA plate and used to inoculate 5 mL of Tryptone Soya Broth medium (TSB, Oxoid, Hampshire, UK), which was subsequently incubated for 24 h at 28 °C to obtain an overnight bacterial culture for further usage.

### 4.7. Assessment of the Antibacterial Properties of PEC-AgNPs and SDS-AgNPs against Dickeya spp. and Pectobacterium spp.

Serial dilutions of 4×, 16×, 64×, 256×, and 1024× of 11.0 mg L^−1^ PEC-AgNPs and 24.0 mg∙L^−1^ SDS-AgNPs stock solutions were prepared in distilled water on the day of the experiment. Two mL of the overnight cultures of Dsol, Pba, Pcbr, Pcc, and Ppa were centrifuged at 6500 rpm for 10 min in order to harvest the bacterial cells. The supernatants were discarded and the pellets were washed twice in sterile 0.85% NaCl to remove residual media. After suspending the pellets in sterile 0.85% NaCl solutions, the densities of the bacterial suspensions were adjusted to 0.5 on the McFarland scale based on DEN-1B densitometer (BioSan, Riga, Latvia) measurements.

MICs for PEC-AgNPs and SDS-AgNPs against Dsol, Pba, Pcbr, Pcc, and Ppa were evaluated in 96-well microplates. In the case of the test samples, each well contained 90 µL of TSB medium, 10 µL of cell suspension at a density of 0.5 in the McFarland scale, and 100 µL of the appropriate PEC-AgNPs or the SDS-AgNPs dilution. Several controls were included in the analysis. The positive controls contained 100 µL sterile water as a substitute for AgNPs. Also, the sterility of the synthesised PEC-AgNPs and SDS-AgNPs, the water used for AgNPs dilutions, the TSB growth medium, and the 0.85% NaCl suspension was tested. Measurements of the absorbance at 600 nm were conducted with the use of an EnVision™ Multilabel Plate Reader (PerkinElmer, Waltham, MA, USA). Afterwards, the microplates were incubated 24 h without shaking at 28 °C. MIC was defined as the lowest concentration of AgNPs inhibiting bacterial growth on the basis of post incubation absorbance measurements at 600 nm. The whole experiment was repeated twice.

MBC was defined as the lowest concentration of PEC-AgNPs and SDS-AgNPs that eliminated 99.9% of the bacterial cells within 24 h. The contents of the microplate wells that showed no bacterial growth were plated on TSA. The appearance of bacterial colonies was assessed after 24 h incubation at 28 °C and was compared to the controls. The whole experiment was performed twice.

## 5. Patents

The method for the synthesis of metallic nanostructures is protected by Polish patent application no. P.417933.

## Figures and Tables

**Table 1:** Antibacterial activity of PEC-AgNPs and SDS-AgNPs against *Dickeya* spp. and *Pectobacterium* spp. Values are based on two replicates, and identical results for each replicate were observed.

Strain	PEC-AgNPs		SDS-AgNPs	
	MIC (mg∙L^−1^)	MBC (mg∙L^−1^)	MIC (mg∙L^−1^)	MBC (mg∙L^−1^)
Dsol IFB0099	5.5	5.5	3.0	3.0
Pba IFB5103	5.5	5.5	0.75	0.75
Pcbr IFB5390	5.5	5.5	3.0	3.0
Pcc IFB5118	5.5	5.5	3.0	3.0
Ppa IFB5308	5.5	5.5	3.0	3.0

**Table 2:** Strains of *Dickeya* and *Pectobacterium* spp. used in this study.

Species	No. IFB UG&MUG Collection	Nos. in Other Collections	Host Plant	Country of Isolation	Reference
*Dickeya solani* (Dsol)	IFB0099	IPO 2276, LMG 28824	*Solanum tuberosum*	Poland, 2005	Slawiak et al. (2009) [59]
*Pectobacterium atrosepticum* (Pba)	IFB5103	SCRI 1086	*Solanum tuberosum*	Canada, 1985	SCRI collection ^b^
*Pectobacterium carotovorum* subsp. *brasiliense* (Pcbr)	IFB5390	LMG21371	*Solanum tuberosum*	Brasil, 2002	Duarte et al. (2004) [60]
*Pectobacterium carotovorum* subsp. *carotovorum* (Pcc)	IFB5118	SCRI 136	*Solanum tuberosum*	USA, NA ^a^	SCRI collection ^b^
*Pectobacterium parmentieri* (Ppa)	IFB5308	SCC3193	*Solanum tuberosum*	Finland, 1980s	Nykyri et al. (2012) [61]

^a^ NA—not available; ^b^ SCRI collection—The James Hutton Institute bacterial collection, Dundee, Scotland.

## Abbreviations

AgNPs	silver nanoparticles
ATR FT-IR	attenuated total reflection-Fourier transformation infrared spectroscopy
CAPP	cold atmospheric pressure plasma
dc-APGD	direct-current atmospheric pressure glow discharge
Dsol	*Dickeya solani*
EDX	energy-dispersive X-ray spectroscopy
FAAS	flame atomic absorption spectrometry
fcc	face cubic centered
FLA	flowing liquid anode
IFB UG&MUG	Intercollegiate Faculty of Biotechnology of the University of Gdansk and Medical University of Gdansk
LSPR	localized surface plasmon resonance
MBC	minimal bactericidal concentration
MIC	minimal inhibitory concentration
NPs	nanoparticles
Pba	*Pectobacterium atrosepticum*
Pcbr	*Pectobacterium carotovorum* subsp. *brasiliense*
Pcc	*Pectobacterium carotovorum* subsp. *carotovorum*
PEC	Pectins
Ppa	*Pectobacterium parmentieri*
ROS	reactive oxygen species
RNS	reactive nitrogen species
SADP	selected area diffraction pattern
SAED	selected area electron diffraction
SDS	sodium dodecyl sulfate
TEM	transmission electron microscopy
TSA	tryptone soya agar
TSB	tryptone soya broth
